# Investigation of the Potential Key Genes and the Multitarget Mechanisms of Polygonum cuspidatum against Heart Failure Based on Network Pharmacology and Experimental Validation

**DOI:** 10.1155/2022/7784021

**Published:** 2022-05-28

**Authors:** Fang Guo, Zhaoqin Xing, Qingwen Sun

**Affiliations:** Department of Cardiology, Shandong Second Provincial General Hospital, Jinan, China

## Abstract

In this study, systematic pharmacology and bioinformatic approaches were employed to identify the potential targets of *Polygonum cuspidatum* (PC) for treating heart failure (HF). The active ingredients of PC were screened by using the TCMSP database, and HF-related genes were identified in the GEO database. Then, the herb-HF targeted-gene networks were constructed using Cytoscape software. Gene Ontology (GO) and Kyoto Encyclopedia of Genes and Genomes (KEGG) functional analyses were performed to obtain the enriched molecular pathways associated with the pathogenesis of HF. Finally, *in vitro* experiment was performed to evidence network pharmacology analysis. 170 intersection genes were obtained, and key genes (FOXO3, NFKB1, and TNF) were identified. Besides, GO and KEGG findings indicated that PC treatment of HF was achieved via regulating apoptosis, IL-17 signaling pathway, TNF signaling pathway, response to oxidative stress, and response to reactive oxygen species. And cell experiment revealed that PC could decrease the expression of NFKB1 and TNF and increase the expression of FOXO3, SOD1, and GPX1 in H9C2 cells. These findings showed that the therapeutic mechanism of PC in the treatment of HF may be associated with the regulation of inflammation-related and oxidative stress-related genes.

## 1. Introduction

Heart failure is a systemic and multifactorial disease, which is defined as the inability of cardiac tissue to provide the peripheral tissues with the oxygen and blood to meet their metabolic needs [1]. It is the final stage of numerous heart diseases, including dilated cardiomyopathy, myocarditis, hypertension, and myocardial infarction. Despite progress in pharmacological treatments, it has high mortality and morbidity and is one of the most important cardiovascular diseases [2]. Therefore, it is important to develop new treatments for the prevention and treatment of HF. HF is considered one of the greatest challenges in cardiovascular disease treatment due to its pathological complexity. Extensive basic research and clinical trials are developing for more effective treatment for HF. An increasing number of reports indicated that inflammation, oxidative stress, and apoptosis are involved in the development of HF and play a vital role in cardiac dysfunction after acute myocardial infarction [3–5]. Therefore, anti-inflammatory, antioxidant, and antiapoptosis therapies may be considered novel therapeutic strategies for HF treatment.

*Polygonum cuspidatum* (PC) is the dried root of *Polygonum cuspidatum* Sieb. et Zucc. and has been used as a traditional medicine in the treatment of various diseases, including hyperlipidemia, skin burns, infection, inflammation, menopausal symptoms, amenorrhea, and chronic bronchitis [6–7]. Phytochemical studies showed that lignans, phenols, coumarins, flavonoids, and anthraquinones are the major active ingredients in PC [8]. Polydatin, an active compound of PC, could treat atherosclerotic diseases via antioxidative stress, regulating lipid metabolism, and inhibiting inflammation [9]. However, little research about the cardioprotective activities of PC is reported. And a deeper investigation of its cardiovascular protective effect and mechanism action is needed.

Due to the multiple ingredients, multiple targets, and multiple pathways of traditional Chinese medicines (TCM), it is difficult to clarify its underlying mechanism actions and identify the active ingredients of TCM [10]. Network pharmacology is an emerging discipline based on the interaction network of “ingredients, targets, disease, and genes,” integrating computational biology, omics, and systems biology to reveal the synergistic effect of multi-ingredients on human diseases through network analysis [11–12]. This method offered a novel sight into the multiple ingredients, multiple targets, and multiple pathways of TCM. Therefore, it is widely used to investigate the underlying mechanism of actions of TCM [13]. To systematically investigate the potential targets and signal pathways of PC in the treatment of HF, we integrated network pharmacology and experimental pharmacology to identify the mechanism of action of PC at the molecular level.

## 2. Materials and Methods

### 2.1. Construction of Active Ingredient Database and Prediction of Potential Targets of PC

The TCMSP database was used to screen the active ingredients of PC based on the following parameters: oral bioavailability (OB) ≥ 30% and drug-likeness (DL) ≥ 0.18. The 2D structures of compounds were obtained from the PubChem database (https://pubchem.ncbi.nlm.nih.gov/). Then, the SDF files of compounds were imported to the SwissTargetPrediction database (http://www.swisstargetprediction.ch/), and the target genes were obtained.

### 2.2. Prediction of HF-Related Genes

The Gene Expression Omnibus dataset (https://www.ncbi.nlm.nih.gov/) was used to download the row gene expression data (GSE59867). Then, the data were processed by the GEO2R tool. The specific information of GSE59867 was presented in supplementary material 1. Finally, the common genes of differentially expressed genes and active ingredients were obtained using the Venny tool (https://bioinfogp.cnb.csic.es/tools/venny/index.html).

### 2.3. PPI Network Construction and Key Gene Identification

The common targets were imported to the STRING database (https://www.string-db.org/), with the confidence score was set 0.9 and the species was set “Homo sapiens” to obtain the PPI network. Then, the TSV file was downloaded and imported to Cytoscape software (3.8.1) to visualize the PPI network. The key genes were selected using cytoHubba plug-in of Cytoscape based on the degree value.

### 2.4. Enrichment Analysis of Target Genes

These common genes were imported to the database (http://metascape.org/gp/#/main/step1) to collect KEGG data and GO biological function data. Then, we used a free online platform (http://www.bioinformatics.com.cn/) to generate the bubble diagrams.

### 2.5. Preparation of PC

The dried root of *Polygonum cuspidatum* Sieb. et Zucc. was obtained from Beijing Pu Sheng Lin Pharmaceutical Co., Ltd. Powdered *Polygonum cuspidatum* was extracted twice with 75% ethanol under reflux. Then, the extract was centrifuged at 3000 × *g* for 5 min, and the supernatant was collected and concentrated under reduced pressure. Finally, the concentrates were lyophilized to obtain PC.

### 2.6. Cell Experiment

H9C2 cells were obtained from China Infrastructure of Cell Line Resources and maintained in Dulbecco's modified Eagle's medium, containing 100 mg/ml streptomycin, 100 U/ml penicillin, and fetal bovine serum (10%) at 37°C with 5% CO2 and 95% air. We changed the medium daily until the H9C2 cells were at 80-90% confluence. H9C2 cells (1 × 10^4^ cell/well) were inoculated into a 96-well plate and then pretreated with different concentrations of PC (50, 100, 200, and 400 *μ*g/ml) for 2 h and then coincubated with angiotensin II (1 *μ*M) for 48 h. Then, a CCK8 Kit was used to measure the cell viability. Briefly, 10 *μ*l of CCK-8 was added to each pore for incubation of 2 h. The absorbance was measured at 450 nm using the microplate reader.

### 2.7. Animal Experiment

The animal care and experimental protocols were approved by the Animal Care Committee of Shandong Second Provincial General Hospital. Male C57BL/6 mice (18-22 g and 8 weeks old) were purchased from the Experimental Animal Center of Shandong Province. All mice were randomly divided into four groups (8 mice/group) and maintained in a pathogen-free laboratory under a 12-hour light/dark cycle and free access to water and chow. The heart failure model was induced by single subcutaneous injections of angiotensin II (1.4 mg/kg/d) for four weeks [14]. Meanwhile, the heart failure model group (angiotensin II) was treated orally with a low dose of PC (LPC, 100 mg/kg/d) or a high dose of PC (HPL, 200 mg/kg/d) every day for four weeks, while the normal group of mice was treated orally with vehicle only. The dose of PC was based on the previous research [15–16].

### 2.8. Assessment of Cardiac Function

Mice were anesthetized with sodium pentobarbital, and shortening score (FS%) and ejection fraction (EF%) were calculated by using the Doppler echocardiography.

### 2.9. Histological Analysis

At the end of the experiment, the mice were euthanized and heart tissues were collected. Parts of cardiac tissues were fixed in 4% paraformaldehyde and then embedded in paraffin. 5 *μ*m thick sections were stained with hematoxylin-eosin reagent based on standard procedure [17].

### 2.10. Western Blot Analysis

We carried out the western blot analysis based on the standard procedure in previous reports [18]. Antibodies for SOD1 (1 : 1000, catalog no.2770), TNF (1: 1000, catalog no.3707), and GAPDH (1: 1000, catalog no.8884) were purchased from Cell Signaling Technology. GAPDH was used as an endogenous control.

### 2.11. Quantitative Real-Time Polymerase Chain Reaction (qPCR)

After drug treatment, total RNA of H9C2 cells was extracted using TRIzol reagent. 2 *μ*g of purified RNA was used to synthesize cDNA by an iScript cDNA synthesis kit (Bio-Rad, USA) based on the manufacturer's protocol. Then, qPCR was performed using SYBR GREEN Premix reagent on the PCR System (Applied Biosystems, CA, USA). The 2^–*ΔΔ*Ct^ method was used to normalize the mRNA expression levels of genes. The sequences of specific primers were shown in Table 1.

### 2.12. Statistical Analysis

Experimental data were shown as mean ± standard deviation (SD). The GraphPad 5 software was used to process the data, and the difference between groups was tested using one-way ANOVA followed by Dunnett's multiple comparison test. *P* < 0.05 was defined as statistically significant.

## 3. Results

### 3.1. Prediction Potential Active Ingredients of PC

As shown in Table 2, we obtained 9 active ingredients of PC via TCMSP retrieval and ADME parameter screening.

### 3.2. Prediction of Targets of PC and HF

**Figure 1:**
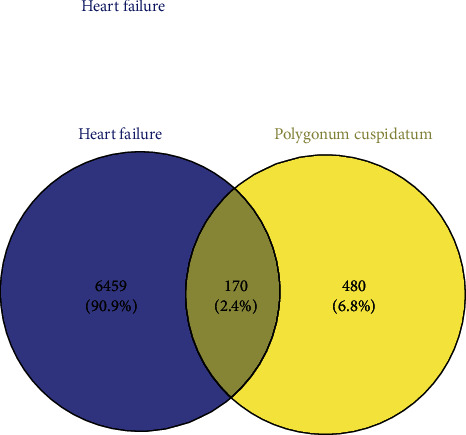
Venn diagram of HF-related disease targets and PC-related targets.

A total of 650 targets of PC were obtained by the SwissTargetPrediction database. A total of 6629 HF-related genes were collected from row gene expression data (GSE59867). Then, the potential genes of the HF and active ingredients targets were imported to the Venny tool analysis platform, and the common genes of HF and PC were screened. As shown in Figure 1, the Venn diagram indicated that 170 potential genes were related to PC and HF.

### 3.3. Construction of PPI Network and Identification of Key Genes

**Figure 2:**
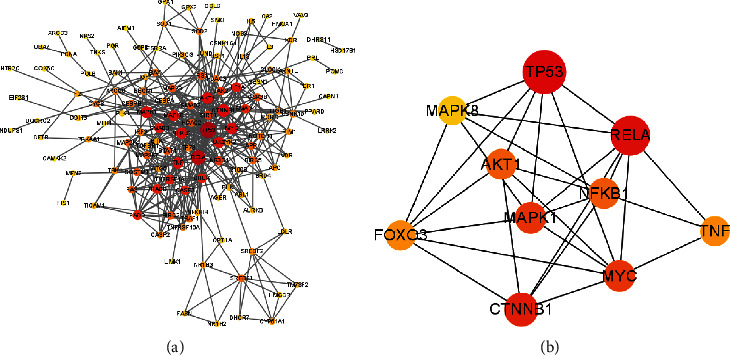
Protein–protein interaction (PPI) network analysis. A network of potential targets of PC with therapeutic effect against HF was visualized using Cytoscape software (a). The top 10 key genes were finally generated by cytoHubba plug-in of Cytoscape (b). Yellow indicates the low degree, while red indicates the high degree.

170 common genes between PC ingredients and HF were imported to the String database, with the highest confidence score of 0.9; then, the PPI network with 133 nodes and 437 edges was visualized via Cytoscape software (Figure 2(a)). We screened the top 10 genes as the key genes, based on the degree value by using cytoHubba plug-in of Cytoscape (Figure 2(b)). It is worth mentioning that FOXO3 and inflammation-related genes (NFKB1 and TNF) were in the top 10. Oxidative stress-related genes, SOD1 and GPX1, are also predicated targets of PC. Thus, we selected these 5 genes for cell experiment verification.

### 3.4. Enrichment Analysis of Common Genes

**Figure 3:**
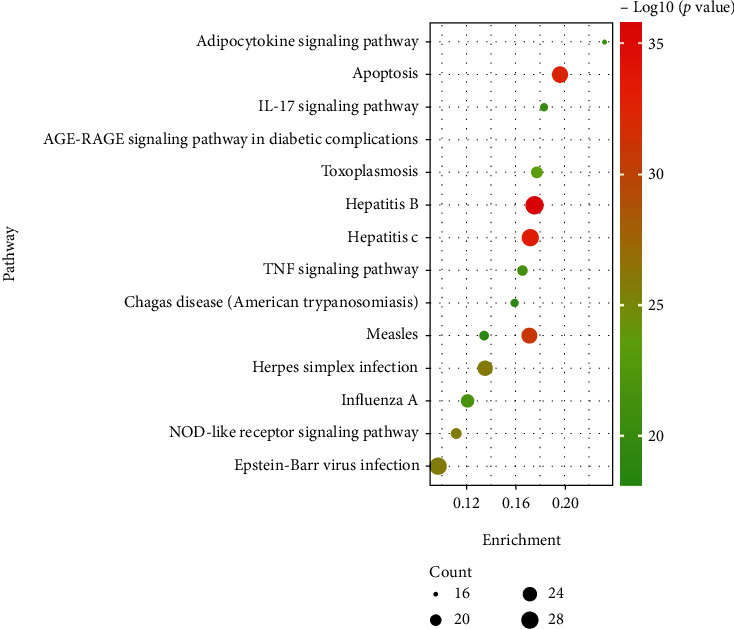
Kyoto Encyclopedia of Genes and Genomes (KEGG) signaling pathway enrichment analysis for potential targets (top 15 were listed).

We carried out the KEGG enrichment analysis to explore which signaling pathways enrich by those target genes and elucidate the underlying mechanism actions of PC against HF. We found that the 170 intersection genes were significantly enriched in 54 signaling pathways according to the results of the KEGG analysis. Among them, the top 15 signaling pathways were selected based on the *p* value (supplement material 2, Table S1). According to the enrichment, gene count, and fold change *p* value, the top 15 signaling pathways were visualized in Figure 3. These results showed that PC exerted a therapeutic effect for HF via integrating multiple signaling pathways, including apoptosis, TNF signaling pathway, and IL-17 signaling pathway.

**Figure 4:**
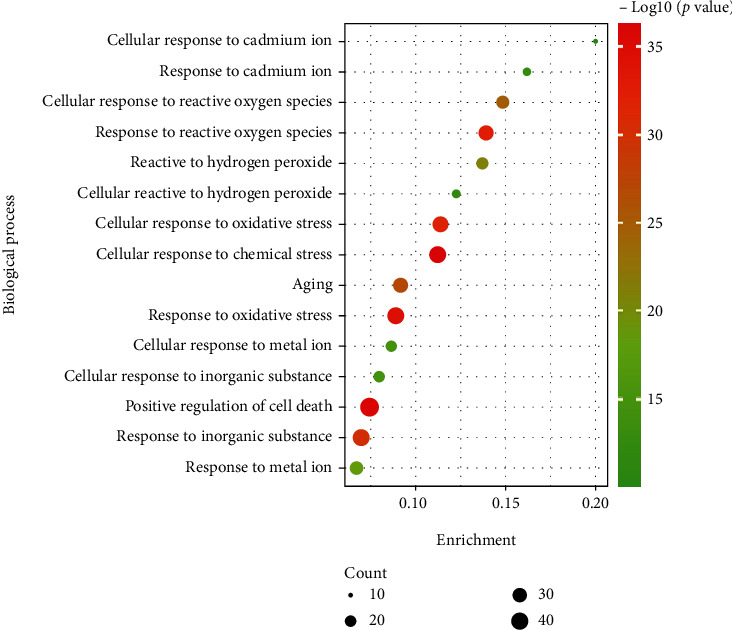
Gene Ontology (GO) biological process enrichment analysis for potential targets (top 15 were listed).

We also performed GO enrichment analysis to investigate the biological function of PC in the treatment of HF. We found that the 170 intersection genes were significantly enriched in 188 GO biological process terms, based on the results of GO analysis. Based on the *p* value, the top 15 GO biological process terms were arranged in supplement material 2 (Table S2). According to the enrichment, gene count, and fold change *p* value, the top 15 GO biological processes were visualized in Figure 4. Moreover, these results revealed that the multibiological processes mainly focused on cellular response to chemical stress, response to oxidative stress, response to reactive oxygen species, cellular response to oxidative stress, response to an inorganic substance, aging, cellular response to reactive oxygen species, and so on.

### 3.5. Analysis of Compound-Gene-Pathway Network

**Figure 5:**
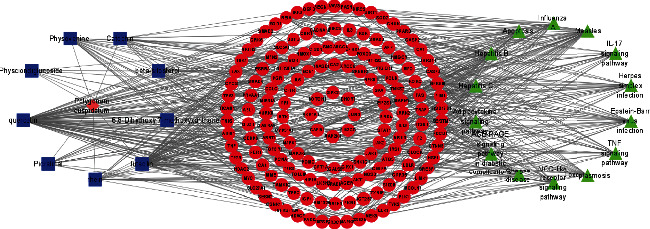
Compound-herb-gene-pathway network. The green triangular nodes indicate the top 14 signaling pathways, the red circular nodes indicate 170 target genes, the blue square nodes represent 9 ingredients, and the other node is PC.

The network with 194 nodes (14 signaling pathways, 9 ingredients, 170 genes, and 1 herb) and 612 edges was visualized in Figure 5. The green triangular nodes indicate the top 14 signaling pathways, the red circular nodes indicate 170 target genes, the blue square nodes represent 9 ingredients, and the other node is PC. Besides, the active ingredients 6,8-dihydroxy-7-methoxyxanthone, physovenine, picralinal, physciondiglucoside, rhein, beta-sitosterol, catechin, luteolin, and quercetin were related to 6, 22, 14, 7, 21, 20, 20, 68, and 90 target genes, respectively. And the signaling pathways apoptosis, TNF signaling pathway, and IL-17 signaling pathway were associated with 27, 19, and 17 target genes, respectively. These results revealed that PC exerted a therapeutic effect for HF via multiple signaling pathways and multiple ingredients with multiple targets.

### 3.6. PC Improved Cell Viability in Angiotensin II-Stimulated H9C2 Cells

**Figure 6:**
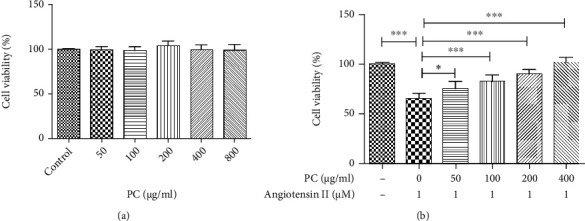
Effect of PC on the cell viability in angiotensin II-stimulated H9C2 cells. H9C2 cells were stimulated with different concentrations of PC (50, 100, 200, 400, and 800 *μ*g/ml) for 48 h (a). H9C2 cells were treated with PC (50, 100, 200, and 400 *μ*g/ml) for 2 h before coculture with angiotensin II (1 *μ*M) for 48 h. (b) CCK-8 method was performed to measure the cell viability. Experimental data were shown as mean ± standard deviation (SD). *n* = 6. ^∗^*P* < 0.05 and ^∗∗∗^*P* < 0.001.

As shown in Figure 6(a), after PC treatment, no significant differences in cell viability were observed for H9C2 cells. This result indicated that PC has no toxic effect on H9C2 cells at the concentrations of 50-800 *μ*g/ml. Besides, treatment with PC (50-400 *μ*g/ml) could significantly improve cell viability in angiotensin II-stimulated H9C2 cells (Figure 6(b)).

### 3.7. The Alleviation of PC on Angiotensin II-Induced Inflammation and Oxidative Stress in H9C2 Cells

**Figure 7:**
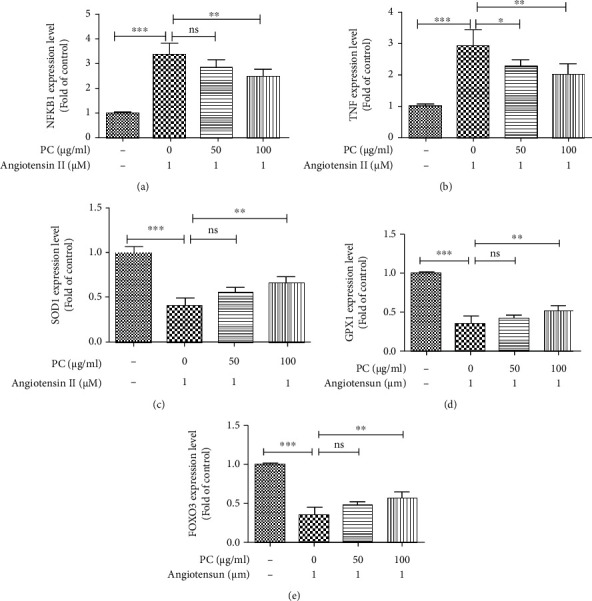
PC attenuation of angiotensin II-induced inflammation and oxidative stress in H9C2 cells. H9C2 cells were treated with PC (50 and 100 *μ*g/ml) for 2 h before coincubation with angiotensin II (1 *μ*M) for 48 h. mRNA expression of NFKB1 (a), TNF (b), SOD1 (c), GPX1 (d), and FOXO3 (e) were measured by qRT-PCR. Experimental data were shown as mean ± standard deviation (SD). *n* = 3. ^∗^*P* < 0.05 and ^∗∗^*P* < 0.01.

The expression levels of NFKB1, TNF, FOXO3, SOD1, and GPX1 in H9C2 cells were measured by qRT-PCR to further validate the network pharmacology prediction of PC in the treatment HF. As shown in Figures 7(a) and 7(b), the expression levels of NFKB1 and TNF were higher in angiotensin II-treated H9C2 cells than in untreated H9C2 cells. PC downregulated the expressions of NFKB1 and TNF in angiotensin II-stimulated H9C2 cells.

Besides, the expression levels of FOXO3, SOD1, and GPX1 were lower in angiotensin II-treated H9C2 cells than in unstimulated H9C2 cells. Treatment with PC upregulated the expressions of FOXO3, SOD1, and GPX1 in angiotensin II-stimulated H9C2 cells (Figures 7(c)–7(e)). These findings indicated that PC could inhibit angiotensin II-induced inflammation and oxidative stress in H9C2 cells.

### 3.8. PC Alleviated Cardiac Dysfunction in Angiotensin II-Treated Mice

**Figure 8:**
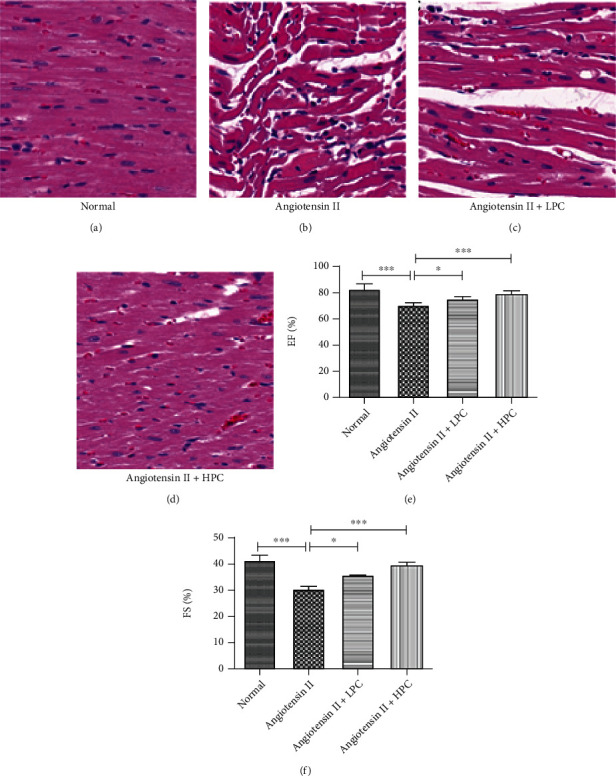
PC alleviated cardiac dysfunction in angiotensin II-treated mice. (a–d) Representative image of H&E staining; (e) ejection fraction (EF); and (f) fractional shortening (FS). Experimental data were shown as mean ± standard deviation (SD). *n* = 6. ^∗^*P* < 0.05, ^∗∗^*P* < 0.01, and ^∗∗∗^*P* < 0.001.

In the present study, the angiotensin II-induced cardiac dysfunction mice were treated with different doses of PC to further confirm the therapeutic effect of PC against heart failure in vivo. Compared with the normal group, the enlarged myofiber cross-sectional area, deranged cellular structures, and disorganized myofibers were observed in cardiac tissues of the angiotensin II group (Figures 8(a) and 8(b)). Both LPC and HPC improved these histopathological changes of the cardiac tissues to varying degrees, especially in the HPC group (Figures 8(c) and 8(d)). Besides, our findings also showed that both LPC and HPC improved angiotensin II-induced decreasing of EF and FS (Figures 8(e) and 8(f)).

### 3.9. PC Alleviated Inflammation and Oxidative Stress in Angiotensin II-Treated Mice

**Figure 9:**
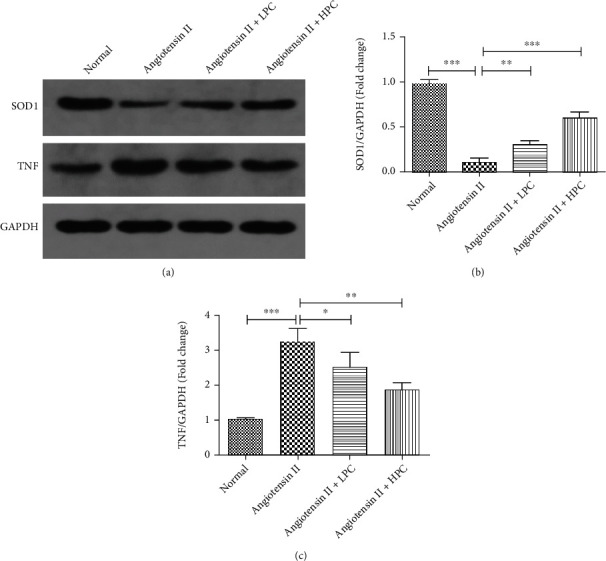
PC alleviated inflammation and oxidative stress in angiotensin II-treated mice. (a) Representative western blot analyses of SOD1, TNF, and GAPDH in cardiac tissues. The normalized optical density of SOD1 (b) and TNF (c). Experimental data were shown as mean ± standard deviation (SD). *n* = 3. ^∗^*P* < 0.05, ^∗∗^*P* < 0.01, and ^∗∗∗^*P* < 0.001.

As shown in Figure 9, western blot analysis showed that both LPC and HPC upregulated protein expression of SOD1 and downregulated protein expression of TNF in angiotensin II-treated mice. These findings revealed that PC could improve angiotensin II-induced oxidative stress and inflammation.

## 4. Discussion

HF is a very common “epidemic” disease in the modern world, with high mortality, morbidity, and hospitalization needs, giving a constant strain on public health systems. To maintain heart function during the development of HF, a complex network of compensatory mechanisms is activated at the molecular and cellular levels [1]. Recent research has revealed that apoptosis, inflammation, and oxidative stress were involved in the development and progression of HF [3–5]. Given that HF involves a variety of pathological processes, drugs that could inhibit inflammation and oxidative stress are potential treatments for chronic HF [19–20]. TCM is such a drug through the action of multiple ingredients, multiple targets, multiple signaling pathways, and multiple physiological functions to play a role in the treatment of diseases.

In the present study, a network pharmacology method was used to screen the active compounds of PC and predict the related potential pharmacological genes and mechanisms. As a result, we predicted 170 pharmacological targets of PC in the treatment of HF, and the resultant top 10 key genes were obtained, including TP53, RELA, CTNNB1, MAPK1, AKT1, NFKB1, FOXO3, MYC, MAPK8, and TNF. Moreover, our GO and KEGG enrichment analyses revealed that PC exerted therapeutic effects against HF via the regulation of inflammation and oxidative stress. Therefore, we chose FOXO3, inflammation-related genes (NFKB1 and TNF), and oxidative stress-related genes (SOD1 and GPX1) to verify the network pharmacological results using cell experiments.

It has been reported that inflammation and immune system activation were involved in the pathogenesis of HF [21]. Recent reports showed that proinflammatory cytokines play an important role in the progression of HF. Proinflammatory cytokines, including IL-6, IL-1*β*, IFN-*γ*, IL-17, and TNF-*α*, could induce cardiac hypertrophy, fibrosis, and apoptosis and further induce inflammation, ultimately resulting in HF. It has been reported that increased TNF-*α* levels were related to ventricular dilatation, cardiac fibrosis, and mortality [22]. NF-*κ*B signaling is a major regulator of inflammation and many other biological processes, such as cell survival and cell growth. Activation of NF-*κ*B could induce the secretion of proinflammatory cytokines, including TNF-*α*, IL-18, and IL-1*β* [23]. Moreover, NF-*κ*B could activate multiple genes to play an important role in the pathogenesis of HF and cardiac hypertrophy [24]. NF-*κ*B activation in cardiomyocytes could lead to HF and cardiomyopathy via stimulating inflammatory responses [25]. The previous report indicated that inhibition of NF-*κ*B could cause regression of cardiac hypertrophy [26]. The YiQiFuMai injection is a TCM and has been shown to improve chronic HF via inactivation of NF-*κ*B and inhibition of proinflammatory cytokine [27]. Kaempferol is a flavonoid compound of medicinal plants and has been shown to alleviate isoproterenol-induced HF via regulation of NF-*κ*B signaling pathway [28]. Therefore, TNF and NF-*κ*B may be the potential therapeutic targets shown to be beneficial in patients with HF. In the present study, we screened TNF and NFKB1 as the key genes in the IL-17 signaling pathway and TNF signaling pathway. Besides, we established the H9C2 cells model of HF to investigate whether PC could decrease the expression levels of TNF and NFKB1. Interestingly, we observed that PC could downregulate the expressions of TNF and NFKB1 in angiotensin II-stimulated H9C2 cells. Thus, our results demonstrated that TNF and NFKB1 were the potential targets of PC in the treatment of HF.

Recent reports have revealed that oxidative stress has attracted some attention as a potential contributor to the progression of HF and a major pathophysiological factor of HF [29–30]. An excessive and deregulated production of reactive oxygen species could trigger further reactive oxygen species generation, which results in the induction of apoptosis, the activation of the inflammatory response, and the stimulation of cardiomyocyte hypertrophy. All of these changes were thought to be major factors in the development of HF [29, 31]. SOD and GPX are the important antioxidant enzymes in the body, which could improve the antioxidant ability to alleviate oxidative stress [32]. FOXO transcription factors are present in cardiac tissue that regulate various biological processes, such as immunity, apoptosis, metabolism, aging, and oxidative stress [33–34]. It has been reported that FOXO3 exerted the cardioprotective effects via inhibition of oxidative stress and upregulation of antioxidant enzyme expressions in paraquat-induced cardiac aging [35]. Angiotensin-(1-7) exerted antihypertrophic effects via decreasing NF-*κ*B levels and increasing SOD1 and FOXO3 in the heart [36]. Besides, Si-Miao-Yong-An decoction is a TCM and has been reported to exert cardioprotective function via restoring the equilibrium of NOX2 and SOD in isoprenaline-induced HF model [37]. Therefore, FOXO3 and SOD1 might be potential therapeutic targets in the treatment of HF. In the present study, FOXO3 was screened as one of the top 10 key genes using the cytoHubba plug-in of Cytoscape. And GO enrichment analysis indicated that PC exerted therapeutic effect against HF via regulation of response to oxidative stress, response to reactive oxygen species, cellular response to oxidative stress, aging, and cellular response to reactive oxygen species. Interestingly, the results of the cell experiment indicated that PC could significantly upregulate the expressions of FOXO3, SOD1, and GPX1 in angiotensin II-stimulated H9C2 cells, implying that FOXO3, SOD1, and GPX1 were the potential targets of PC against HF.

## 5. Conclusions

In the present study, the network pharmacology and cell experimental validation were performed to validate the therapeutic effects and mechanism actions of PC in the treatment of HF. The therapeutic mechanism of PC against HF might be associated with the upregulation of oxidative stress-related genes and inhibition of inflammation-related genes. Our findings also provided directions for exploring the mechanism actions and potential targets of PC for the treatment of HF.

## Figures and Tables

**Table 1:** Sequences of primers used quantitative real-time PCR.

Gene	Forward primer (5′ to 3′)	Reverse primer (5′ to 3′)
TNF	CCCTGGTATGAGCCCATCTATC	AAAGTAGACCTGCCCAGACTCG
NFKB1	ACACCGTGTAAACCAAAGCC	CAGCCAGTGTTGTGATTGCT
FOXO3	CAGATCTACGAGTGGATGGTG	TCTTGCCAGTTCCCTCATTC
SOD1	CCGGCTTGTCTGATGGAGAT	TGCATCTTTTGGTCCACCGT
GPX1	TGAGAAGTGCGAGGTGAATG	CGGGGACCAAATGATGTACT
GAPDH	TGGTGGACCTCATGGCCTAC	CAGCAACTGAGGGCCTCTCT

**Table 2:** The active ingredients of PC.

MOL_ID	Molecule name	MW	OB (%)	DL	Molecular structure
MOL013281	6,8-Dihydroxy-7-methoxyxanthone	258.24	35.83	0.21	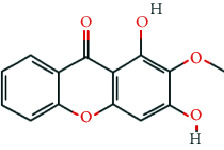
MOL013287	Physovenine	262.34	106.21	0.19	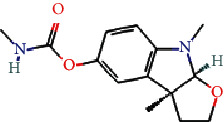
MOL013288	Picralinal	366.45	58.01	0.75	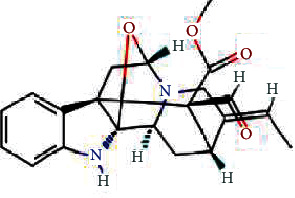
MOL002259	Physciondiglucoside	608.6	41.65	0.63	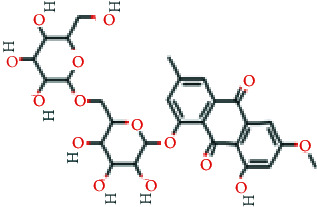
MOL002268	Rhein	284.23	47.07	0.28	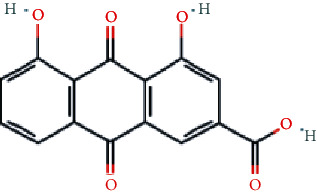
MOL000358	Beta-sitosterol	414.79	36.91	0.75	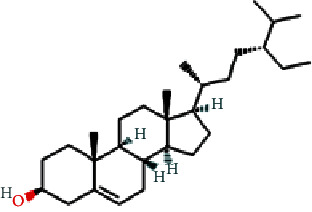
MOL000492	(+)-Catechin	290.29	54.83	0.24	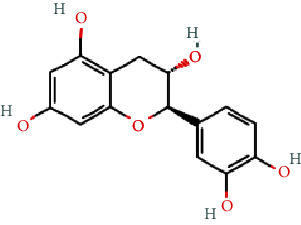
MOL000006	Luteolin	286.25	36.16	0.25	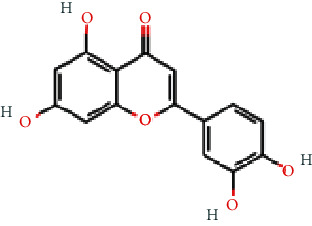
MOL000098	Quercetin	302.25	46.43	0.28	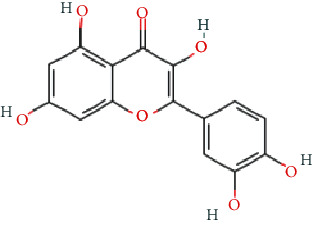

## Data Availability

The data used to support the study are available from the corresponding author.

## Supplementary Materials

Supplementary Materials Table S1 KEGG enrichment analysis of PC for HF (top 15). Table S2 GO enrichment analysis of PC for HF (top 15).
