# Supplementary material for: Investigation of the Potential Key Genes and the Multitarget Mechanisms of Polygonum cuspidatum against Heart Failure Based on Network Pharmacology and Experimental Validation
Source: Dis Markers. 2022 May 28;2022:7784021. doi: 10.1155/2022/7784021 (PMC9167087; doi:10.1155/2022/7784021)
Supplement: Supplementary Materials — Table S1 KEGG enrichment analysis of PC for HF (top 15). Table S2 GO enrichment analysis of PC for HF (top 15). [file 7784021.f1.pdf]

Table S1 KEGG enrichment analysis of PC for HF (top 15).

Term	Pathway	Enrichment	p value	Genes
hsa05161	Hepatitis B	0.1751	1.59E-36	AKT1, BIRC5, FAS, BAX, CASP3, CHUK, ELK1, FASN, IRF3, JAK2, SMAD2, SMAD3, SMAD4, MYC, NFKB1, PCNA, MAPK1, MAPK8, MAPK9, RELA, MAP2K4, STAT1, MAP3K7, TGFBR1, TNF, TP53, TYK2, FADD, TBK1, CYCS, TICAM1
hsa05160	Hepatitis C	0.1716	6.43E-34	AKT1, FAS, BAK1, BAX, CASP3, CHUK, CTNNB1, EIF2S1, FASN, GSK3B, IRF3, LDLR, MYC, NFKB1, MAPK1, MAPK8, MAPK9, RELA, STAT1, TNF, TNFRSF1A, TP53, TYK2, TRADD, FADD, NR1H3, TBK1, CYCS, TICAM1
hsa04210	Apoptosis	0.1957	2.88E-33	AKT1, BIRC2, BIRC5, FAS, BAK1, BAX, CAPN1, CASP2, CASP3, CHUK, DDIT3, EIF2S1, IL3, NFKB1, MAPK1, MAPK8, MAPK9, RELA, TNF, TNFRSF1A, TP53, TRAF1, TRADD, FADD, TNFRSF10A, AIFM1, CYCS
hsa05162	Measles	0.1709	1.46E-31	AKT1,FAS,BAK1,BAX,CASP3,CHUK,EIF2S1,FASN,GSK3B,IL13,IRF3,JAK2,NFKB1,NFKBIB,MAPK8,MAPK9,RELA,STAT1,MAP3K7,TP53,TP73,TYK2,TRADD,FADD,TNFRSF10A,TB
hsa05168	Herpes simplex infection	0.1351	1.33E-26	FAS, ARNTL, CASP3, CHUK, EIF2S1, IRF3, JAK2, NFKB1, NFKBIB, PER1, MAPK8, MAPK9, RELA, STAT1, MAP3K7, TNF, TNFRSF1A, TP53, TRAF1, TYK2, FADD, CLOCK, TBK1, CYCS, TICAM1
hsa05169	Epstein-Barr virus infection	0.0970	1.64E-26	AKT1, FAS, BAK1, BAX, BTK, CASP3, CHUK, FASN, GSK3B, HDAC2, IRF3, MYC, NFKB1, NFKBIB, MAPK8, MAPK9, RELA, RNASE1, MAP2K4, STAT1, MAP3K7, TNF, TP53, TRAF1, TYK2, TRADD, FADD, TBK1, CYCS

ko05145	Toxoplasmosis	0.1770	6.42E-24	AKT1, BIRC2, CASP3, CHUK, ITGB1, JAK2, LDLR, NFKB1, NFKBIB, PIK3CG, MAPK1, MAPK8, MAPK9, RELA, STAT1, MAP3K7, TNF, TNFRSF1A, TYK2, CYCS
hsa04933	AGE-RAGE signaling pathway in diabetic complications	0.1776	8.40E-23	AGER, AKT1, BAX, CASP3, JAK2, SMAD2, SMAD3, SMAD4, NFKB1, NOS3, PIM1, MAPK1, MAPK8, MAPK9, RELA, RNASE1, STAT1, TGFBR1, TNF
hsa05164	Influenza A	0.1209	2.04E-22	AKT1, FAS, EIF2S1, FASN, GSK3B, IRF3, JAK2, NFKB1, NFKBIB, MAPK1, MAPK8, MAPK9, RELA, MAP2K4, STAT1, TNF, TNFRSF1A, TYK2, TNFRSF10A, TBK1, CYCS, TICAM1
hsa04668	TNF signaling pathway	0.1652	3.60E-22	AKT1, BIRC2, FAS, CASP3, CEBPB, CHUK, FASN, NFKB1, MAPK1, MAPK8, MAPK9, RELA, MAP2K4, MAP3K7, TNF, TNFRSF1A, TRAF1, TRADD, FADD
ko04920	Adipocytokine signaling pathway	0.2319	2.20E-21	AKT1, CHUK, CPT1A, IRS1, JAK2, NFKB1, NFKBIB, POMC, PRKAA1, MAPK8, MAPK9, RELA, TNF, TNFRSF1A, TRADD, CAMKK2
ko04657	IL-17 signaling pathway	0.1828	9.63E-21	CASP3, CEBPB, CHUK, GSK3B, IL5, IL13, JUND, NFKB1, MAPK1, MAPK8, MAPK9, RELA, MAP3K7, TNF, TRADD, FADD, TBK1
hsa05142	Chagas disease (American trypanosomiasis)	0.1589	1.21E-19	AKT1, FAS, CHUK, FASN, SMAD2, SMAD3, NFKB1, MAPK1, MAPK8, MAPK9, RELA, MAP2K4, TGFBR1, TNF, TNFRSF1A, FADD, TICAM1

ko05162	Measles	0.1343	2.27E-19	AKT1, FAS, CHUK, EIF2S1, GSK3B, IL13, IRF3, JAK2, NFKB1, NFKBIB, RELA, STAT1, MAP3K7, TP53, TP73, TYK2, TNFRSF10A, TBK1
hsa04621	NOD-like receptor signaling pathway	0.1118	7.74E-19	BIRC2, CHUK, IRF3, NFKB1, NFKBIB, MAPK1, MAPK8, MAPK9, RELA, STAT1, MAP3K7, TNF, TYK2, FADD, ATG5, MFN2, TXNIP, TBK1, TICAM1

Table S2 GO enrichment analysis of PC for HF (top 15).

Term	GO function	Enrichment	p value	Genes
GO:0062197	cellular response to chemical stress	0.1121	4.25E-37	ABL1, AKT1, FAS, ARNTL, BTK, CASP3, CHUK, CTNNB1, DDIT3, EIF2S1, FOXO3, G6PD, XRCC6, GPX1, HDAC2, HIF1A, HMOX1, HSF1, JAK2, MPO, NOS3, PCNA, PRKAA1, MAPK1, MAPK8, MAPK9, RELA, SOD1, SOD2, TP53, BECN1, AIFM1, ATG5, CAMKK2, SIRT1, SESN2, LRRK2, MIR34A, APP, BAK1, GCLC, GPX2, STAT1, TXNIP, CA2, CEBPA, HSD17B1, JUND, KDR, SLC5A1, FIS1, MCOLN1, AGER, CASP2, HMGCR, MME, POLB, MAP2K4, SREBF1, AURKB, NEK6, PDCD4
GO:0006979	response to oxidative stress	0.0889	5.10E-35	ABL1, AKT1, APP , ARNTL, BAK1, BTK, CASP3, CHUK, CTNNB1, EIF2S1, FOXO3, G6PD, GCLC, GPX1, GPX2, HDAC2, HIF1A, HMOX1, HSF1, JAK2, MPO, NOS3, PCNA, PRKAA1, MAPK1, MAPK8, MA
GO:0000302	response to reactive oxygen species	0.1390	3.00E-33	ABL1, AKT1, BAK1, BTK, CASP3, CHUK, FOXO3, GPX1, HDAC2, HIF1A, HMOX1, HSF1, MPO, NOS3, PCNA, PRKAA1, MAPK1, MAPK8, MAPK9, RELA, SOD1, SOD2, STAT1, BECN1, AIFM1, TXNIP, CAMKK2, SIRT1, SESN2, LRRK2, MIR34A

GO:0034599	cellular response to oxidative stress	0.1138	1.99E-32	ABL1, AKT1, ARNTL, BTK, CHUK, CTNNB1, EIF2S1, FOXO3, G6PD, GPX1, HDAC2, HIF1A, HMOX1, HSF1, JAK2, MPO, NOS3, PCNA, PRKAA1, MAPK1, MAPK8, MAPK9, RELA, SOD1, SOD2, TP53, BECN1, AIFM1, CAMKK2, SIRT1, SESN2, LRRK2, MIR34A
GO:0010035	response to inorganic substance	0.0697	7.25E-31	ABL1, AKT1, APP, BAK1, CA2, CASP3, CEBPA, CHUK, EIF2S1, FOXO3, G6PD, GCLC, GPX1, HDAC2, HIF1A, HMOX1, HSD17B1, HSF1, JUND, KDR, MPO, NOS3, PCNA, PRKAA1, MAPK1, MAPK8, MAPK9, RELA, SLC5A1, SOD1, SOD2, STAT1, BECN1, AIFM1, TXNIP, SIRT1, FIS1, MCOLN1, LRRK2, MIR34A
GO:0007568	aging	0.0914	1.62E-27	ABL1, AGER, AKT1, APP, ARNTL, BAK1, CASP2, EIF2S1, FOXO3, GCLC, HMGCR, JUND, MME, MPO, POLB, MAPK1, MAPK8, MAPK9, RELA, MAP2K4, SOD1, SOD2, SREBF1, TP53, BECN1, AURKB, NEK6, SIRT1, PDCD4, LRRK2, MIR34A
GO:0034614	cellular response to reactive oxygen species	0.1484	1.65E-25	ABL1, AKT1, BTK, CHUK, FOXO3, HDAC2, HSF1, MPO, NOS3, PCNA, PRKAA1, MAPK1, MAPK8, MAPK9, RELA, SOD1, SOD2, BECN1, AIFM1, CAMKK2, SIRT1, LRRK2, MIR34A
GO:0042542	response to hydrogen peroxide	0.1370	1.39E-21	ABL1, BAK1, CASP3, FOXO3, GPX1, HDAC2, HMOX1, HSF1, PCNA, PRKAA1, RELA, SOD1, SOD2, STAT1, BECN1, AIFM1, TXNIP, SIRT1, LRRK2, MIR34A
GO:0010038	response to metal ion	0.0670	5.12E-19	AKT1, APP, CA2, CASP3, CEBPA, CHUK, EIF2S1, G6PD, GCLC, HIF1A, HMOX1, HSD17B1, HSF1, JUND, PCNA, PRKAA1, MAPK1, MAPK8, MAPK9, SOD1, SOD2, BECN1, TXNIP, MCOLN1, LRRK2
GO:0071241	cellular response to inorganic substance	0.0796	2.84E-15	AKT1, APP, CEBPA, CHUK, EIF2S1, HMOX1, SD17B1, HSF1, JUND, PRKAA1, MAPK1, MAPK8, MAPK9, SOD1, BECN1, AIFM1, MCOLN1, LRRK2

GO:0071248	cellular response to metal ion	0.0863	4.57E-15	AKT1, APP, CEBPA, CHUK, EIF2S1, HMOX1, HSD17B1, HSF1, JUND, PRKAA1, MAPK1, MAPK8, MAPK9, SOD1, BECN1, MCOLN1, LRRK2
GO:0046686	response to cadmium ion	0.1618	3.19E-13	AKT1, CHUK, GCLC, HMOX1, HSF1, PCNA, MAPK1, MAPK8, MAPK9, SOD1, SOD2
GO:0070301	cellular response to hydrogen peroxide	0.1224	8.20E-13	ABL1, FOXO3, HDAC2, HSF1, PCNA, PRKAA1, RELA, BECN1, AIFM1, SIRT1, LRRK2, MIR34A
GO:0071276	cellular response to cadmium ion	0.2000	9.75E-11	AKT1, CHUK, HMOX1, HSF1, MAPK1, MAPK8, MAPK9, SOD1
GO:0010942	positive regulation of cell death	0.0744	6.76E-37	ABL1, AKT1, APC, FAS, BAK1, BAX, CASP2, CASP3, CTNNB1, DDIT3, EIF2S1, ELK1, FOXO3, GSK3B, HMGCR, HMOX1, ITGB1, JAK2, LGALS9, NOTCH1, MAPK8, MAPK9, S100B, MAP2K4, SFRP1, SOD1, SOD2, TGFBR1, TNF, TNFRSF1A, TP53, TP73, VDR, BECN1, TRADD, FADD, TNFRSF10A, SQSTM1, AIFM1, MFN2, TXNIP, SIRT1, PDCD4, FIS1, LRRK2, MIR34A, BTK, CEBPB, GCLC, GPX1, HIF1A, IGF1, SMAD3, MLH1, NOS3, POLB, PPARD, RELA, TRAF1, TICAM1, APP, G6PD, GABRA5, HSF1, IL13, TBK1
